# Prevention of Football Injuries

**DOI:** 10.5812/asjsm.34869

**Published:** 2010-06

**Authors:** Donald T Kirkendall, Astrid Junge, Jiri Dvorak

**Affiliations:** FIFA Medical Assessment and Research Center (F-MARC), Schulthess Clinic, Lengghalde 2, 8008 Zürich, Switzerland

**Keywords:** Sport Injury, Football, Systematic Review, Prevention

## Abstract

**Purpose:**

Every sport has a unique profile of injury and risk of injury. In recent years, there have been numerous attempts at conducting injury prevention trials for specific injuries or for injuries within specific sports to provide evidence useful to the sports medicine and sport community. Football has been a focus of a number of randomized injury prevention trials.

**Methods:**

MEDLINE was searched with the first order keywords of “injury prevention” and “sport”. This list was restricted to “clinical trial” or “randomized controlled trial” which had been conducted on children and adults whose goal was preventing common football injuries. Our objective was to find studies with an exercise-based training program, thus projects that used mechanical interventions were excluded.

**Results:**

A structured, generalized warm-up has been shown to be effective at preventing common injuries in football, reducing injuries by about one-third.

**Conclusion:**

The huge participation numbers in the worldwide family of football would suggest that any reduction in injury should have a public health impact. Professionals in sports medicine need to promote injury prevention programs that have been shown to be effective.

## INTRODUCTION

Football is without question the world's most popular sport with an estimated 265 million registered players^[1]^. Much of the current growth is due to the rapid increase in the number of females playing as well as the growth in countries where football does not have a strong historical record such as the United States, China, and India^[2]^. Sport carries with it the risk of injury and each sport has its own particular injury profile. Any increase in participation within a sport will be accompanied by an increase in the number of injuries. Any increase in injuries in a sport with the participation numbers like football will likely have a public health impact in terms of the burden on health care systems as well as time lost to education and productivity.

One pillar of the professional sports medicine community is injury prevention and while the medical community has been a visible presence in sport, the emphasis on prevention historically has been based on logic and expert opinion. For example, static stretching has long been considered as a practice that prevents muscle strain injury, but has come under increased scrutiny recently^[3–5]^. Thus, both the medical and sporting communities are looking less at what practices would seem to make sense and more at programs that are supported by evidence based on data derived from clinical trials.

The model for sports injury prevention research follows a conceptual process described by van Mechelen^[6]^. This 4–step model begins by determining the incidence of injury, determining the mechanism of each injury to be prevented, designing and implementing prevention interventions, and finally reassessing the injury incidence to see if the intervention was successful or not. In practice, a large group of athletes or teams are randomly assigned to either a control group or an intervention group. Injuries for a full season are recorded and the exposure-related injury rates between the two groups are compared.

In the 1980's, Ekstrand and colleagues^[7–10]^ published the results of the first injury prevention trials in professional football. It was not until the mid to late 1990's that prevention trials were conducted on a wider scale. These trials were of two types: trials to prevent a specific injury or those designed to prevent a wider spectrum of injuries. As ankle sprain is one of the most common injuries in sport, a number of studies have been published whose goal was to reduce the incidence of ankle sprain^[11–24]^. The goal of other projects was to prevent other common injuries such as tendon injury^[25]^, hamstring strains^[26–30]^, groin strains^[31, 32]^, and knee sprains – the anterior cruciate ligament in particular^[33–39]^. Other studies were designed to decrease a broad range of common injuries. Many of these more broad-based projects replaced a traditional warm-up with a generalized warm-up that consisted of activities to reduce common injuries in that particular sport. In football, as with most team sports, the most common injuries are ligament sprains (of the ankle and knee) and muscle strains (of the hamstring and groin). After considering the mechanisms of injury and the activities shown to be successful in preventing specific injuries, researchers design generalized warm-up programs based on the best available evidence. The results of these generalized warm-up programs will be presented here.

## SEARCH METHODS

MEDLINE was searched with the first order keywords of “injury prevention” and “sport” (n = 3,018). This list was restricted to “clinical trial” or “randomized controlled trial” (n = 212) which had been conducted on “all children (0–18)” and “adults (19–44)” (n = 179) whose goal was preventing common football injuries (ankle sprain, knee sprains, muscle strains). Each title and abstract was reviewed to confirm the use of random assignment and that injuries were compared between the control group and the intervention group. Our objective was to find studies with an exercise-based training program, thus projects that used mechanical interventions (such as taping, bracing, or other orthoses) were excluded. As football was the focus of the current study, only trials on football players were selected.

## RESULTS AND DISCUSSION

We identified 18 prevention trials on football players, ten injury-specific and eight generalized injury prevention projects. Table 1 summarizes the results of these 18 projects. The generalized programs will be discussed.

**Table 1:** Summary of injury-specific and generalized injury prevention trials in football

Author	Focus	Gender	Age	Purpose	Groups	Intervention	Outcomes
**McGuine**^[17]^	ankle	both	high school	prevent ankle injuries football and basketball players	373 intervention and 393 controls	5-phase balance board program with eyes open or closed on the floor (phases 1-2) then on a balance board(phases 3-5)	Significant reduction in the incidence of ankle sprain in the intervention group. Significantly lower in the intervention group.
**Ohammadi**^[20]^	ankle	male	adult	prevent recurrent ankle sprains in football players	random assignment of 80 players into 1 of 3 study groups or control	strength training, orthosis, proprioceptive training	Significantly fewer sprains in the proprioceptive group vs. the control group
**Caraffa**^[33]^	knee	male	adult	prevent ACL injury in football players	600 intervention and 300 control players	proprioceptive training program using diferent types of wobble boards	Intervention group had significantly fewer ACL injuries
**Hewett**^[35]^	kne	female	high school	prevent ACL injuries female athletes	43 sport teams from 12 schools. 366 intervention, 463 controls, 434 control males	preseason progressive jumptraining program (60-90min/day x 3d/wk)	Significantly lower relative incidence of serious knee injury in the intervention group. Intervention group had no non-contact knee ligament injuries in soccer or baskeball players.
**Mandlebaum**^[37]^	knee	female	high school	prevent ACL injuries in football players	2 year total enrollment: 1885 intervention, 3818 controls	guided warmup consisting of stretching, strengthening, plyometrics, agility exercises	Significant reductions in ACL injury each year. 88% reductionin year 1 and 74% reduction in year 2.
**Pfeiffer**^[39]^	knee	female	high school	prevent ACL injury football players	577 intevention and 862 controls	twice per week in-season plyometric exercises	ACL injury rates were not different between groups
**Gilchrist**^[34]^	knee	female	college	prevent ACL injury in football players	583 intervention and 852 controls	guided warm-up program of exercises for neuromuscular control	Significant reduction in recurrent ACL injury. 41% fewer ACL tears, 70% fewer non-contact ACL tears.
**Askling**^[27]^	muscle	male	adult	prevent hamstrig strains professional football players	30 players chosen from teams Each randomly placed to a hamstring strenghening program or control	eccentric overload of the hamstrings	Significantly fewer hamstrings inthe intervention group (3/15) vs the control group (10/15).
**Homlich**^[31]^	muscle	male	adult	Prevent goin strain in professional football players	524 intervention 453 controls	exercises for muscles about the pelvis to increas strength, cordination, and core stability	Intervention group had 31% fewer groin strains(n.s.). Injury risk increased with a history of a prior strain and with increasing level of play.
**Fredberg**^[25]^	muscle-tendon	male	adult	prevent muscle-tendon injury to the patellar and Achilles tendons in football players	209 players on 12 teams.Random assignment of teams to intervention or control	stretchig and eccentric strengthening	No differences in injury rates. Intervenion reduced the ultrasonic evidence of abnormal tissue.
**Ekstrand**^[9]^	overall	male	adult	prevent injury inprofessional football players	12 teams (180 players),randomly assigned to controlor intervention groups	therapist supervision of a multifactorial programme of improved training and equipment	75% reduction in injuries by players in the intervention group
**Soderman**^[41]^	overall	female	adult	prevent lower extremity injuries in football players	Random assignment of volunteers. 62 intervention, 78 control	10-15 minutes of balance board exercises and normal soccer training	No differences in injury type or incidence. Poor compliance
**Heidt**^[43]^	overall	female	high school	prevent injury in football players	random assignment of players. 42 intervention, 258 control	7 week supervised preseason speed and agility training program	Significantly lower incidence in the intervention group(14% vs. 34%)
**Junge**^[44]^	overall	male	high school	prevent injuries in football players	194 intervention, 101 controls	exercises for core stability, lower extremity strength, neuromuscular control, agility	36% fewer injuries in intervention group. Greatest effect in low skilled players. Fewer mild, overuse, and training injuries.
**Hagglund**^[45]^	overall	male	high school- adult	prevent reinjury in amateur football players	random assignment of teams.216 intervention, 221 control	10-step progressive rehabilitation program and education (risk factors, rehabilitation principles)	75% reduction in lower extremity reinjury risk in intervention teams
**Engebretsen**^[40]^	overall	male	adult	prevent re-injury to the ankle, knee, hamstring, or groin in professional football players	high risk intervention n = 193, high risk control n = 195, low risk control n = 12Q	exercises for core stability, proprioception balance, strength	No difference in injury incidence or severity, poor compliance
**Steffen**^[42]^	overall	female	high school	prevent injury in football players	random assignment of teams. 1091 intervention, 1001 control	agility, neuromuscular control, core stability, lower extremity strength exercises	No difference in injury rate, poor compliance
**Soligard**^[49]^	overall	female	high school	prevent injury in football players	random assignment of teams.1055 intervention players, 837 control players	progressive warmup program of strength, balance, neuromuscular control, core stability exercises	significant reductions in rates of overall (-32%), overuse (-43%),severe (-45%) injuries in the intervention players

**Prevention of Specific Injuries:** There are a number of projects that provide evidence about reducing specific injuries. For example, the incidence of ankle sprains has been reduced by 30–35% using balance board^[14, 17]^ or proprioceptive training^[15, 20]^. While strengthening programs have resulted in reductions in hamstring strains^[27]^, the 30% reduction in groin strains reported by Tyler et al ^[32]^ was not statistically significant. Impressive reductions in ACL injury, with an 88% or greater reduction in ACL tears, have been reported^[36, 37]^. Studies that failed to find a reduction in ligament injury rates have poor compliance to the intervention program^[40–42]^.

**Randomized Trials to Prevent Football Injuries:** There have been attempts at developing a more generalized warm-up or preseason-training program that have met with variable success. The prevention program used in Ekstrand's early work^[8–10]^ on male Swedish professionals was very comprehensive with a multitude of interventions that included specific prevention exercises, training modification, and changes in equipment that were all supervised by the medical staff. An impressive 75% reduction in all injuries was achieved. With such a expansive approach to prevention, however, it was hard to differentiate, for example, whether the ankle exercises or the protective bracing had the greatest role in the reduction of ankle sprains^[9]^. This project provided the first evidence that supplemental programs could have a role in preventing injury in football players.

Randomized trials for preventing football injuries were infrequent until 2000 when Soderman and colleagues^[41]^ published their results of a balance board program on 221 female adult players. The program consisted of 10–15 minutes of balance board training added to their normal training and was an attempt to duplicate the positive findings of Caraffa et al^[33]^ Unfortunately, the raw incidence of severe anterior cruciate ligament injury was greater in the intervention group, although the difference was not statistically significant. An issue was the large number of dropouts and poor compliance of the subjects with the program.

As stated earlier, generalized injury prevention programs are based mostly on exercises from programs shown to prevent specific injuries. A different approach was taken by Heidt and co-workers 43 who evaluated the value of a pre-season fitness program on potential subsequent injury rates. High school female football players (n = 300, 14–18 years of age) were followed for one year (an August-September high school season and their March-August club season). Prior to the club season, 42 players were randomly chosen to follow a 7-week training program that emphasized on endurance, plyometrics, flexibility, and resistance trainings designed to improve speed and agility of the subjects. Other players performed their usual preparations and served as a control group. The type and location of time loss injuries were recorded for both groups. Of the trained players, 14% sustained an injury while 34% of the control group was injured (p = 0.008). There were a total of 32 knee injuries with only three occurring to players in the intervention group. Only one player in the intervention group had a season ending injury while there were 11 season-ending injuries to players in the control group. Unfortunately, while injury incidence was documented, exposure was not collected so rates and risks were unreported.

Junge et al^[44]^ designed the first generalized program for young males, age 14–19, of varied skill levels. The program was made up of 10 exercises to improve endurance, reaction time, coordination, lower extremity stability, plus flexibility and strength of the trunk, hip, and lower extremities. Weekly injury reports were collected over an entire year. Overall, there were 36% fewer injuries per player in the intervention group (p < 0.01). Other significant reductions included nearly 50% fewer mild injuries, 41% fewer overuse injuries, 55% fewer training injuries, and 80% fewer groin injuries. In addition, the players with the highest skill had the lowest injury rates. This was one of the first projects to demonstrate benefits of a generalized injury prevention program. The success of this program was the inspiration for a number of more recent publications.

Hagglund and co-workers^[45]^ took a slightly different approach. Probably the main risk factor for most injuries is a history of that particular injury^[46–48]^. So this project, in which 20 fourth division amateur teams of Swedish males participated, was an attempt to prevent a recurrent injury in football players. All players documented their personal injury history. Players in the intervention teams received information about risk factors of re-injury and principles of rehabilitation in addition to a 10-step rehabilitation program. The program consisted of nine progressive running and sport-specific exercises and return to play criteria that were based on the severity of the injury. The coach was the controlling figure for the teams using the intervention program. Overall, there was a 66% reduction in re-injury risk as a result of the program. The authors also tracked the time between the index injury and a re-injury and reported that the intervention was most effective at preventing a re-injury in the first week of return to play.

Engebretsen et al^[40]^ focused their attention on professional players with an injury history. Each player's injury history was determined in order to establish whether a player was at a high or a low risk of re-injury. The high-risk players were randomized to a control or intervention group while the low risk players also served as a separate control group. Injury specific progressive programs were designed for players with a high risk of an ankle, knee, groin, or hamstring injury. Programs were merged for players who were at a high risk of multiple injuries. The interventions were performed three times a week for the 10-week preseason period, then once per week for the remainder of the competitive season. As expected, the low-risk control players had a significantly lower injury incidence than the high-risk players. There were no differences in overall or specific injuries between the high-risk control and high-risk intervention groups. Compliance in the intervention group was poor; only 19-29% of the players completed 30 or more training sessions. A total of 16%, 23%, 63% and 68% of the players at high risk for an ankle, knee, hamstring, and groin injury respectively, reported no exercise sessions. This project is a further illustration of the importance of complying with a prevention program.

A generalized exercise program based on the original work of Junge et al^[44]^ was developed by the medical research program of FIFA and was called “The 11”. A cluster-randomized trial of “The 11” was conducted by Steffen and colleagues^[42]^ on female youth players (14–18yr of age). A total of 113 teams (2,092 players) were randomized into a control or intervention group where the intervention teams were instructed regarding “The 11”. This 15–minute warm-up program was to be used by the intervention teams throughout the eight-month season. There were no differences between groups for either the overall injury rate or the rates for individual injuries. Compliance was again a limitation. For the first half of the season, the training program was used during 60% of the training sessions, but by the end of the season, less than one in four teams had performed 20 sessions.

**Fig. 1:**
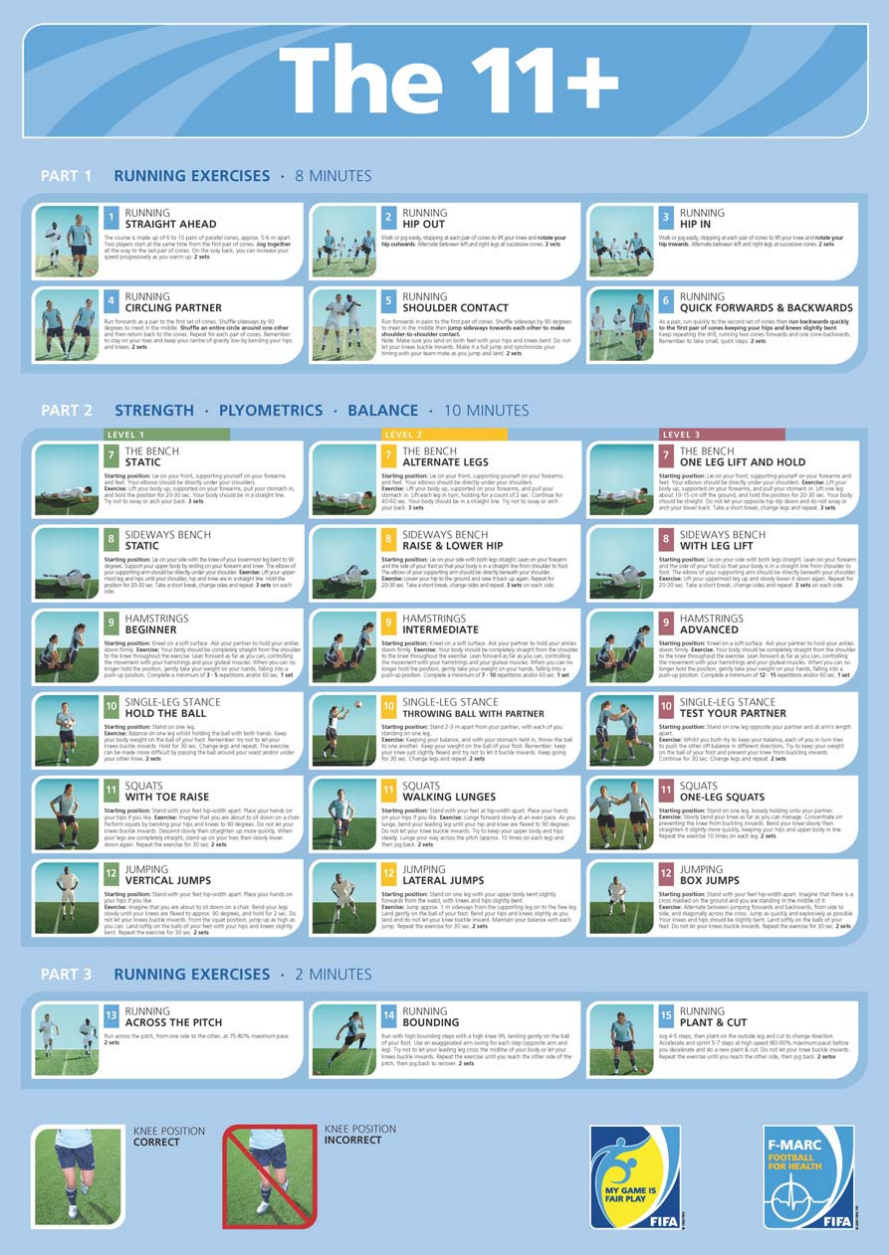
Poster of the 11+. Freely available at extranet.fifa.com/medical

As researchers began to see some limitations with earlier programs (e.g. lack of progression of exercises), FIFA's Medical Assessment and Research Centre (F-MARC) in cooperation with the research groups at the Oslo Sports Trauma Research Centre and the Santa Monica Orthopaedic and Sports Medicine Research Foundation developed a three-part generalized warm-up^[49]^. Part 1 is a series of six slow-speed running exercises that should take about eight minutes to complete. Part 2 includes six exercises of strength, plyometric, and balance activities. Each of these six exercises has three levels of progression that challenges players as their ability and capacity improves. This part of the warm-up should take about 10 minutes. Part 3 concludes the warm-up with three more running exercises that are more demanding than those in Part 1. The increased intensity of work brings the player closer to the running demands of the formal football training to follow. This final portion should take only about two minutes. Once the players learn the exercises, the entire warm-up program should take about 20 minutes. This does not increase the duration of training as this program substituted for whatever warm-up program was being conducted. The program was termed *The 11 + * (Fig. 1).

Details of the program are as follows; the warm-up area for Part 1 is two parallel lines of 6-10 cones set about five to six meters apart. Players would go through the course in pairs with each successive pair of players starting when the pair in front has left the second cone. When finishing each exercise, the player jogs back to the starting point. The jogging speed of the return leg can increase progressively as each player warms-up.

### Part 1: Running Exercises

This part takes about 8 minutes to complete. Each exercise is done twice and in pairs.

**Straight ahead:** Players jog through the course to the last pair of cones.

**Hip out:** Players walk or jog easily, stop at each cone, lift their knee and **rotate the hip outward**. Alternate between left and right legs at successive cones.

**Hip in:** Players walk or jog easily, stop at each cone, lift their knee and **rotate the hip inward**. Alternate between left and right legs at successive cones.

**Circling partner:** Players run forward to the first set of cones – shuffle sideways 90 degrees inwards and meet in the middle – **shuffle an entire circle around each other** – and then return back to the cones. Repeat for each pair of cones. Players should remember to stay on their toes, keep their centre of gravity low, and bend at the hips and knees.

**Shoulder contact:** Players run forward to the first pair of cones – shuffle sideways 90 degrees and meet in the middle – **jump sideways towards each other making shoulder to shoulder contact. Note:** Players should land on both feet with the knees bent. The knees should not buckle inwards. Players should make it a full jump and synchronize the timing with their teammate as they jump and land.

**Quick forwards and backwards:** Players run quickly to the second set of cones. **Run backwards quickly to the previous cone keeping their hips and knees slightly bent –** keep repeating this drill running two cones forward and one cone backwards. Remember, small, quick steps.

### Part 2: Strength, Plyometrics and Balance

Many of these require a partner. Each exercise has three progressively more challenging levels. Begin with level 1 of each exercise. As the player's fitness improves for any individual exercise, move to its level 2. Doing one level of each exercise should take about 10 minutes.

**The Bench**

**Level 1: Static**

**Starting position:** Players lie on their front, supported on their forearms and both feet. Their elbows should be directly under their shoulders.

**Exercise:** Players lift their body up, supported on their forearms. They should pull their stomach in and hold the position for 20–30 seconds. Their body should be in a straight line without sway or an arched back; 3 sets.

**Level 2: Alternate legs**

**Starting position:** Players lie on their front, supported on their forearms and both feet. Their elbows should be directly under their shoulders.

**Exercise:** Players lift their body up, supported on their forearms. They should pull their stomach in and alternately lift each leg repeatedly – holding for a count of 2 seconds. Continue for 40–60 seconds. Their body should be in a straight line without sway or an arched back; 3 sets.

**Level 3: One leg lift and hold**

**Starting position:** Players lie on their front, supported on their forearms and both feet. Their elbows should be directly under their shoulders.

**Exercise:** Players lift their body up, supported on their forearms. They should pull their stomach in. Lift one leg about 10–15 cm off the ground and hold the position for 20–30 seconds. Their body should be straight. They should not let the opposite hip dip down, nor should they sway or arch their low back. Let them take a short break, change legs and repeat; 3 sets.

**Sideways Bench**

**Level 1: Static**

**Starting position:** Players lie on one side with the knee of the lowermost leg bent to 90 degrees. Their body is supported by resting on the forearm and knee. The elbow of their support arm should be directly under their shoulder.

**Exercise:** Players lift their uppermost leg and hips until their shoulder, hip, and knee are in a straight line. Hold the position for 20–30 seconds. After a short break, change sides and repeat; 3 sets on each side.

**Level 2: Raise and Lower Hip**

**Starting position:** Players lie on their side with both legs straight. The players should lean on their forearm and the side of their foot so they are in a straight line from shoulder to foot. The elbow of their support arm should be directly under their shoulder.

**Exercise:** Players lower their hip down to the ground and raise it back up again. Repeat for 20–30 seconds. After a short break, change sides and repeat; 3 sets on each side.

**Level 3: With leg lift**

**Starting position:** Players lie on their side with both legs straight. The players should lean on their forearm and the side of their foot so they are in a straight line from shoulder to foot. The elbow of their support arm should be directly under their shoulder.

**Exercise:** Players lift the uppermost leg and slowly lower it back down again. Repeat the exercise for 20–30 seconds. After a short break, change sides and repeat; 3 sets on each side.

**Hamstrings**

**Level 1: Beginner**

**Starting position:** Players kneel on a soft surface while a partner firmly holds the ankles down.

**Exercise:** Players need to keep their body completely straight from shoulders to knees throughout the exercise. They should lean forward as far as possible controlling the movement with their hamstrings and gluteal muscles. When they can no longer hold the position, they gently take their weight on their hands, falling into a push-up position. Do a minimum of 3–5 repetitions or 60 seconds; 1 set.

**Level 2: Intermediate**

Players perform the same exercise for a minimum of 7-10 repetitions or 60 seconds; 1 set.

**Level 3: Advanced**

Players perform the same exercise for a minimum of 12–15 repetitions or 60 seconds; 1 set.

**Single Leg Stance**

**Level 1: Hold the ball**

**Starting position:** Players stand on one leg.

**Exercise:** Players balance on one leg whilst holding a ball in their hands. Their weight should be on the ball of their foot. The knee should not buckle inwards. Hold this position for 30 seconds, then change legs and repeat. This exercise can be made more difficult by passing the ball around their middle and/or under the opposite knee; 2 sets.

**Level 2: Throwing Ball with Partner**

**Starting position:** Two players stand 2–3 meters apart with each standing on one leg.

**Exercise:** With players maintaining their balance and their stomach held in, they throw the ball to one another. Their weight should be on the ball of their foot. Their knee should be just slightly flexed and not allowed to buckle inwards. Keep going for 30 seconds. Change legs and repeat; 2 sets.

**Level 3: Test Your Partner**

**Starting position:** Partners stand on one leg facing each other at arm's length apart.

**Exercise:** With players trying to maintain their balance, each player in turn tries to push their partner off balance in different directions. Their weight should be on the ball of their foot; they should prevent their knee from buckling inwards. Keep going for 30 seconds. Change feet and repeat; 2 sets.

**Squats**

**Level 1: With Toe Raise**

**Starting position:** Players stand with their feet hip-width apart. They can place their hands on their hips if they like.

**Exercise:** Players should imagine they are about to sit down on a chair. Perform squats by bending their hips and knees to 90 degrees without letting their knees buckle inwards. Descend slowly and straighten up more quickly. When their knees are completely straight, they should stand up on their toes and then slowly lower down again. Repeat the exercise for 30 seconds; 2 sets.

**Level 2: Walking Lunges**

**Starting position:** Players stand with their feet hip-width apart. They can place their hands on their hips if they like.

**Exercise:** Players should lunge forward slowly at an even pace. As they lunge forward, the lead leg should bend until the hip and knee are flexed to 90 degrees. The knees should not buckle inwards. Keeping the upper body and hips steady, the players should work their way across the pitch (approx. 10 times on each leg) and then jog back; 2 sets.

**Level 3: One-Leg Squats**

**Starting position:** Partners stand side by side on one leg loosely holding onto their partner.

**Exercise:** Both players slowly bend their knee as far as manageable. Players need to concentrate on preventing the knee from buckling inwards. They should bend their knee slowly and straighten it slightly more quickly, keeping the hips and upper body in line. Repeat this exercise 10 times on each leg; 2 sets on each leg.

**Jumping**

**Level 1: Vertical Jumps**

**Starting position:** Players stand with their feet hip-width apart. They can place their hands on their hips if they like.

**Exercise:** Players should imagine they are about to sit down on a chair. They should bend their legs slowly until their knees are flexed to approx. 90 degrees and hold for 2 seconds. Do not allow the knees to buckle inwards. From this squat position, they jump as high they can, landing softly on the balls of their feet, and bending their hips and knees. Repeat for 30 seconds; 2 sets.

**Level 2: Lateral Jumps**

**Starting position:** Players stand on one leg with their upper body bent slightly forward with their knees and hips slightly bent.

**Exercise:** Each player should jump approximately 1 meter sideways from the supporting leg on to the free leg. They should land gently on the ball of their foot, bending the hips and knee slightly while not letting the knee buckle inwards. They should maintain balance with each jump. Repeat this exercise for 30 seconds; 2 sets.

**Level 3: Box Jumps**

**Starting position:** Players stand with their feet hip-width apart. They should imagine there is a cross marked on the ground and they are standing in the middle of it.

**Exercise:** Players should alternate jumping forwards and backward, from side to side, and diagonally across the cross. They should jump as quickly and explosively as possible. Their knees and hips should be slightly bent. The players should land softly on the ball of their feet and not let the knees buckle inwards. Repeat the exercise for 30 seconds; 2 sets.

### Part 3 – Running Exercises

These final running exercises take about 2 minutes to complete.

**Across the Pitch:** Players run across the pitch, from one side to the other, at 75–80% of maximum pace; 2 sets.

**Bounding:** Players should run with high bounding steps with a high knee lift, landing gently on the ball of the foot. They should use an exaggerated arm swing for each step (opposite arm and opposite leg). Their lead leg should not cross their midline nor should their knee buckle inwards. Repeat the exercise until they reach the other side of the pitch, then jog back to recover; 2 sets.

**Plant and Cut:** Players jog 4–5 steps then plant on the outside leg and cut to change direction. Players accelerate and sprint 5–7 steps at high speed (80–90% maximum pace) before decelerating and moving into the next plant and cut. Their knee should not buckle inwards. Repeat the exercise until they reach the other side of the pitch and then jog back; 2 sets.

A total of 125 clubs of females, aged 13–17 years of age, were randomized into either a control or an intervention group. *The 11 + * was to be performed at each training session and parts 1 and 3 only were to be performed on match days. Since 13 clubs in the intervention group and 19 clubs in the control group were excluded, the final sample consisted of 52 intervention clubs (n=1,055) and 41 control clubs (n=837). The average age for both groups was 15.4 years of age. The individual player dropout rate was 2.1% and 2.9% for the intervention and control groups, respectively.

In the study, the coach was to ensure correct performance, posture, and good body control by monitoring the players. The coach had to make certain that each player controlled their knee position during the exercises, including straight leg alignment, knee-over-toe position and soft landings. The knee should never buckle inwards into what would appear to be a valgus position.

A total of 16% of all players sustained 376 injuries, 80% of which were acute and 20% were overuse. There were 161 injuries in the intervention group and 215 injuries in the control group.

The rate ratio of the risk of injury was used to determine the risk of injury in the groups. Consistent with other studies, there was a 32% reduction in overall injury risk in the intervention group (*P*=0.04). Match injury risk was reduced by 28% (*P*=0.05), training injury risk was reduced by 32% (*P*=0.01) and lower extremity injury risk was reduced by 29% (p = 0.07). The risk of overuse injury was reduced by 53% (*P*=0.01) and the risk of serious injury (defined as >28 days lost) was reduced by 45% (*P*=0.05).

When specific body parts or injury types were examined, the risk of knee injury was 45% less (*P*=0.005), the risk of contusions was reduced by 56% (*P*=0.007), the risk of lower extremity tendon pain was reduced by 52% (*P*=0.047), and the risk of low back pain was reduced by 89% (*P*=0.04). There was a significant 49% reduction in the risk of multiple injuries. Many other outcome variables trended toward a reduced risk of injury, but did not reach statistical significance when adjusted for the clustered sampling.

The importance of compliance has been a factor in the success of other prevention trials. With individualized instruction, communication between the research team and the clubs combined with the expanded set of progressive exercises and the running activities, a compliance rate of 77% was achieved. The authors felt that, “… the overall rate of injuries, as well as the rate of match injuries, training injuries, overuse injuries, and acute injuries differed significantly. The rate of severe injuries, severe overuse injuries, and severe acute injuries was significantly lower in the intervention group”^[49]^. Incorporating exercises for core stability, strength, and balance with neuromuscular control of the hip and knee for proper movement biomechanics and awareness about the improving motor control helped reduce the risk of all injuries by about one-third and of severe injury by about one-half.

While the evidence supporting the effectiveness of a generalized program continues to grow, the results are not limited to football as successful general injury and specific injury prevention trials have been conducted on team handball^[38, 50]^, floorball^[51]^, basketball^[12, 16]^, volleyball^[11]^, and Australian Rules Football^[30]^.

## CONCLUSION

The results of recent injury prevention trials show that a generalized, structured and progressive warm-up is effective at reducing injury in football. While there are injury-specific protocols, a generalized warm-up program like *The 11 + * is an effective injury prevention program. As the evidence from successful intervention trials continues to mount, the sports medicine community needs to be conscientious in promoting prevention as a primary educational effort. The coach on the field needs to be encouraged to implement injury prevention to ensure the player's health and continued development as a football player.

## References

[1] FIFA (2010). The Big Count. http://www.fifa.com/worldfootball/bigcount/index.html.

[2] Kuper S, Szymanski S (2009). Soccernomics: Why England Loses, Why Germany and Brazil Win, and Why the U.S., Japan, Australia, Turkey--and Even Iraq--Are Destined to Become the Kings of the World's Most Popular Sport.

[3] Shrier I (1999). Stretching before exercise does not reduce the risk of local muscle injury: a critical review of the clinical and basic science literature. Clin J Sports Med.

[4] Witvrouw E, Mahieu N, Danneels L (2004). Stretching and injury prevention: an obscure relationship. Sports Med.

[5] Woods K, Bishop P, Jones E (2007). Warm-up and stretching in the prevention of muscular injury. Sports Med.

[6] van Mechelen W (1997). Sports injury surveillance systems.'One size fits all'?. Sports Med.

[7] Ekstrand J (1982). The frequency of muscle tightness and injuries in soccer players. Am J Sports Med.

[8] Ekstrand J, Gillquist J (1983). The avoidability of soccer injuries. Int J Sports Med.

[9] Ekstrand J, Gillquist J, Liljedahl SO (1983). Prevention of soccer injuries. Supervision by doctor and physiotherapist. Am J Sports Med.

[10] Ekstrand J, Gillquist J, Möller M (1983). Incidence of soccer injuries and their relation to training and team success. Am J Sports Med.

[11] Bahr R, Lian O, Bahr IA (1997). A twofold reduction in the incidence of acute ankle sprains in volleyball after the introduction of an injury prevention program: a prospective cohort study. Scand J Med Sci Sports.

[12] Emery CA, Rose MS, McAllister JR (2007). A prevention strategy to reduce the incidence of injury in high school basketball: a cluster randomized controlled trial. Clin J Sports Med.

[13] Handoll HH, Rowe BH, Quinn KM, de Bie R (2001). Interventions for preventing ankle ligament injuries. Cochrane Database Syst Rev.

[14] Holme E, Magnusson SP, Becher K (1999). The effect of supervised rehabilitation on strength, postural sway, position sense and re-injury risk after acute ankle ligament sprain. Scand J Med Sci Sports.

[15] Hupperets MD, Verhagen EA, van Mechelen W (2009). Effect of unsupervised home based proprioceptive training on recurrences of ankle sprain: randomised controlled trial. Brit Med J.

[16] Kofotolis N, Kellis E (2007). Ankle sprain injuries: a 2-year prospective cohort study in female Greek professional basketball players. J Athl Train.

[17] McGuine TA, Keene JS (2006). The effect of a balance training program on the risk of ankle sprains in high school athletes. Am J Sports Med.

[18] McHugh MP, Tyler TF, Mirabella MR (2007). The effectiveness of a balance training intervention in reducing the incidence of noncontact ankle sprains in high school football players. Am J Sports Med.

[19] Mickel TJ, Bottoni CR, Tsuji G (2006). Prophylactic bracing versus taping for the prevention of ankle sprains in high school athletes: a prospective, randomized trial. J Foot Ankle Surg.

[20] Mohammadi F (2007). Comparison of 3 preventive methods to reduce the recurrence of ankle inversion sprains in male soccer players. Am J Sports Med.

[21] Sitler M, Ryan J, Wheeler B (1994). The efficacy of a semirigid ankle stabilizer to reduce acute ankle injuries in basketball. A randomized clinical study at West Point. Am J Sports Med.

[22] Verhagen E, van Tulder M, van der Beek AJ (2005). An economic evaluation of a proprioceptive balance board training programme for the prevention of ankle sprains in volleyball. Br J Sports Med.

[23] Verhagen EA, van der Beek AJ, Twisk J (2004). The effect of a proprioceptive balance board training program for the prevention of ankle sprains: a prospective controlled trial. Am J Sports Med.

[24] Wester JU, Jespersen SM, Nielsen KD (1996). Wobble board training after partial sprains of the lateral ligaments of the ankle: a prospective randomized study. Phys Ther.

[25] Fredberg U, Bolvig L, Andersen NT (2008). Prophylactic training in asymptomatic soccer players with ultrasonographic abnormalities in Achilles and patellar tendons: the Danish Super League Study. Am J Sports Med.

[26] Arnason A, Andersen TE, Holme I (2008). Prevention of hamstring strains in elite soccer: an intervention study. Scand J Med Sci Sports.

[27] Askling C, Karlsson J, Thorstensson A (2003). Hamstring injury occurrence in elite soccer players after preseason strength training with eccentric overload. Scand J Med Sci Sports.

[28] Brooks JH, Fuller CW, Kemp SP, Redding DB (2006). Incidence, risk, and prevention of hamstring muscle injuries in professional rugby union. Am J Sports Med.

[29] Croisier JL, Ganteaume S, Binet J (2008). Strength imbalances and prevention of hamstring injury in professional soccer players: a prospective study. Am J Sports Med.

[30] Gabbe BJ, Branson R, Bennell KL (2006). A pilot randomised controlled trial of eccentric exercise to prevent hamstring injuries in community–level Australian Football. J Sci Med Sport.

[31] Holmich P, Larsen K, Krogsgaard K, Gluud C (2009). Exercise program for prevention of groin pain in football players: a cluster randomized trial. *Scand J Med Sci Sports*.

[32] Tyler TF, Nicholas SJ, Campbell RJ (2002). The effectiveness of a preseason exercise program to prevent adductor muscle strains in professional ice hockey players. Am J Sports Med.

[33] Caraffa A, Cerulli G, Projetti M (1996). Prevention of anterior cruciate ligament injuries in soccer, A prospective controlled study of proprioceptive training. Knee Surg Sports Traumatol Arthrosc.

[34] Gilchrist J, Mandelbaum BR, Melancon H (2008). A randomized controlled trial to prevent noncontact anterior cruciate ligament injury in female collegiate soccer players. Am J Sports Med.

[35] Hewett TE, Lindenfeld TN, Riccobene JV (1999). The effect of neuromuscular training on the incidence of knee injury in female athletes. A prospective study. Am J Sports Med.

[36] Kiani A, Hellquist E, Ahlqvist K (2010). Prevention of soccer-related knee injuries in teenaged girls. Arch Int Med.

[37] Mandelbaum BR, Silvers HJ, Watanabe DS (2005). Effectiveness of a neuromuscular and proprioceptive training program in preventing anterior cruciate ligament injuries in female athletes: 2-year follow-up. Am J Sports Med.

[38] Myklebust G, Engebretsen L, Braekken IH (2007). Prevention of noncontact anterior cruciate ligament injuries in elite and adolescent female team handball athletes. Inst Course Lect.

[39] Pfeiffer RP, Shea KG, Roberts D (2006). Lack of effect of a knee ligament injury prevention program on the incidence of noncontact anterior cruciate ligament injury. J Bone Joint Surg Am.

[40] Engebretsen AH, Myklebust G, Holme I (2008). Prevention of injuries among male soccer players: a prospective, randomized intervention study targeting players with previous injuries or reduced function. Am J Sports Med.

[41] Söderman K, Werner S, Pietilä T (2000). Balance board training: prevention of traumatic injuries of the lower extremities in female soccer players? A prospective randomized intervention study. Knee Surg, Sports Traumatol Arthrosc.

[42] Steffen K, Myklebust G, Olsen OE (2008). Preventing injuries in female youth football--a cluster-randomized controlled trial. Scand J Med Sci Sports.

[43] Heidt RS, Sweeterman LM, Carlonas RL (2000). Avoidance of soccer injuries with preseason conditioning. Am J Sports Med.

[44] Junge A, Rösch D, Peterson L (2002). Prevention of soccer injuries: a prospective intervention study in youth amateur players. Am J Sports Med.

[45] Hägglund M, Waldén M, Ekstrand J (2007). Lower reinjury rate with a coach-controlled rehabilitation program in amateur male soccer: a randomized controlled trial. Am J Sports Med.

[46] Dvorak J, Junge A (2000). Football injuries and physical symptoms. A review of the literature. Am J Sports Med.

[47] Dvorak J, Junge A, Chomiak J (2000). Risk factor analysis for injuries in football players. Possibilities for a prevention program. Am J Sports Med.

[48] Faude O, Junge A, Kindermann W (2007). Risk factors for injuries in elite female soccer players. Br J Sports Med.

[49] Soligard T, Myklebust G, Steffen K (2008). Comprehensive warm-up programme to prevent injuries in young female footballers: cluster randomised controlled trial. *Br Med J*.

[50] Olsen OE, Myklebust G, Engebretsen L (2005). Exercises to prevent lower limb injuries in youth sports: cluster randomised controlled trial. Br Med J.

[51] Pasanen K, Parkkari J, Pasanen M (2008). Neuromuscular training and the risk of leg injuries in female floorball players: cluster randomised controlled study. *Br Med J*.

